# Age-specific effects of hemoglobin A1c, blood pressure, and cholesterol levels on incident cardiovascular diseases among adults with diabetes in China: a 10-year prospective cohort study

**DOI:** 10.1093/lifemeta/loag008

**Published:** 2026-04-03

**Authors:** Yue Yin, Xiaojing Jia, Shengli Wu, Hong Qiao, Guijun Qin, Tiange Wang, Chunyan Hu, Hong Lin, Shuangyuan Wang, Yu Xu, Mian Li, Min Xu, Jie Zheng, Xiadi He, Yingfen Qin, Xulei Tang, Zhen Ye, Ruying Hu, Lixin Shi, Qing Su, Xuefeng Yu, Li Yan, Qin Wan, Gang Chen, Zhengnan Gao, Guixia Wang, Feixia Shen, Xuejiang Gu, Zuojie Luo, Li Chen, Xinguo Hou, Yanan Huo, Qiang Li, Yinfei Zhang, Tianshu Zeng, Chao Liu, Youmin Wang, Tao Yang, Huacong Deng, Lulu Chen, Jiajun Zhao, Yiming Mu, Guang Ning, Yuhong Chen, Jieli Lu, Weiqing Wang, Yufang Bi

**Affiliations:** Department of Endocrine and Metabolic Diseases, Shanghai Institute of Endocrine and Metabolic Diseases, Ruijin Hospital, Shanghai Jiao Tong University School of Medicine, Shanghai 200025, China; Shanghai National Clinical Research Center for Metabolic Diseases, Key Laboratory for Endocrine and Metabolic Diseases of the National Health Commission of the P.R. China, Shanghai Key Laboratory for Endocrine Tumor, Shanghai National Center for Translational Medicine, Ruijin Hospital, Shanghai Jiao Tong University School of Medicine, Shanghai 200025, China; Department of Endocrine and Metabolic Diseases, Shanghai Institute of Endocrine and Metabolic Diseases, Ruijin Hospital, Shanghai Jiao Tong University School of Medicine, Shanghai 200025, China; Shanghai National Clinical Research Center for Metabolic Diseases, Key Laboratory for Endocrine and Metabolic Diseases of the National Health Commission of the P.R. China, Shanghai Key Laboratory for Endocrine Tumor, Shanghai National Center for Translational Medicine, Ruijin Hospital, Shanghai Jiao Tong University School of Medicine, Shanghai 200025, China; Department of Endocrine and Metabolic Diseases, Karamay Municipal People’s Hospital, Karamay, Xinjiang 834000, China; Department of Endocrine and Metabolic Diseases, The Second Affiliated Hospital of Harbin Medical University, Harbin, Heilongjiang 150086, China; Department of Endocrine and Metabolic Diseases, The First Affiliated Hospital of Zhengzhou University, Zhengzhou, Henan 450052, China; Department of Endocrine and Metabolic Diseases, Shanghai Institute of Endocrine and Metabolic Diseases, Ruijin Hospital, Shanghai Jiao Tong University School of Medicine, Shanghai 200025, China; Shanghai National Clinical Research Center for Metabolic Diseases, Key Laboratory for Endocrine and Metabolic Diseases of the National Health Commission of the P.R. China, Shanghai Key Laboratory for Endocrine Tumor, Shanghai National Center for Translational Medicine, Ruijin Hospital, Shanghai Jiao Tong University School of Medicine, Shanghai 200025, China; Department of Endocrine and Metabolic Diseases, Shanghai Institute of Endocrine and Metabolic Diseases, Ruijin Hospital, Shanghai Jiao Tong University School of Medicine, Shanghai 200025, China; Shanghai National Clinical Research Center for Metabolic Diseases, Key Laboratory for Endocrine and Metabolic Diseases of the National Health Commission of the P.R. China, Shanghai Key Laboratory for Endocrine Tumor, Shanghai National Center for Translational Medicine, Ruijin Hospital, Shanghai Jiao Tong University School of Medicine, Shanghai 200025, China; Department of Endocrine and Metabolic Diseases, Shanghai Institute of Endocrine and Metabolic Diseases, Ruijin Hospital, Shanghai Jiao Tong University School of Medicine, Shanghai 200025, China; Shanghai National Clinical Research Center for Metabolic Diseases, Key Laboratory for Endocrine and Metabolic Diseases of the National Health Commission of the P.R. China, Shanghai Key Laboratory for Endocrine Tumor, Shanghai National Center for Translational Medicine, Ruijin Hospital, Shanghai Jiao Tong University School of Medicine, Shanghai 200025, China; Department of Endocrine and Metabolic Diseases, Shanghai Institute of Endocrine and Metabolic Diseases, Ruijin Hospital, Shanghai Jiao Tong University School of Medicine, Shanghai 200025, China; Shanghai National Clinical Research Center for Metabolic Diseases, Key Laboratory for Endocrine and Metabolic Diseases of the National Health Commission of the P.R. China, Shanghai Key Laboratory for Endocrine Tumor, Shanghai National Center for Translational Medicine, Ruijin Hospital, Shanghai Jiao Tong University School of Medicine, Shanghai 200025, China; Department of Endocrine and Metabolic Diseases, Shanghai Institute of Endocrine and Metabolic Diseases, Ruijin Hospital, Shanghai Jiao Tong University School of Medicine, Shanghai 200025, China; Shanghai National Clinical Research Center for Metabolic Diseases, Key Laboratory for Endocrine and Metabolic Diseases of the National Health Commission of the P.R. China, Shanghai Key Laboratory for Endocrine Tumor, Shanghai National Center for Translational Medicine, Ruijin Hospital, Shanghai Jiao Tong University School of Medicine, Shanghai 200025, China; Department of Endocrine and Metabolic Diseases, Shanghai Institute of Endocrine and Metabolic Diseases, Ruijin Hospital, Shanghai Jiao Tong University School of Medicine, Shanghai 200025, China; Shanghai National Clinical Research Center for Metabolic Diseases, Key Laboratory for Endocrine and Metabolic Diseases of the National Health Commission of the P.R. China, Shanghai Key Laboratory for Endocrine Tumor, Shanghai National Center for Translational Medicine, Ruijin Hospital, Shanghai Jiao Tong University School of Medicine, Shanghai 200025, China; Department of Endocrine and Metabolic Diseases, Shanghai Institute of Endocrine and Metabolic Diseases, Ruijin Hospital, Shanghai Jiao Tong University School of Medicine, Shanghai 200025, China; Shanghai National Clinical Research Center for Metabolic Diseases, Key Laboratory for Endocrine and Metabolic Diseases of the National Health Commission of the P.R. China, Shanghai Key Laboratory for Endocrine Tumor, Shanghai National Center for Translational Medicine, Ruijin Hospital, Shanghai Jiao Tong University School of Medicine, Shanghai 200025, China; Department of Endocrine and Metabolic Diseases, Shanghai Institute of Endocrine and Metabolic Diseases, Ruijin Hospital, Shanghai Jiao Tong University School of Medicine, Shanghai 200025, China; Shanghai National Clinical Research Center for Metabolic Diseases, Key Laboratory for Endocrine and Metabolic Diseases of the National Health Commission of the P.R. China, Shanghai Key Laboratory for Endocrine Tumor, Shanghai National Center for Translational Medicine, Ruijin Hospital, Shanghai Jiao Tong University School of Medicine, Shanghai 200025, China; Department of Endocrine and Metabolic Diseases, Shanghai Institute of Endocrine and Metabolic Diseases, Ruijin Hospital, Shanghai Jiao Tong University School of Medicine, Shanghai 200025, China; Shanghai National Clinical Research Center for Metabolic Diseases, Key Laboratory for Endocrine and Metabolic Diseases of the National Health Commission of the P.R. China, Shanghai Key Laboratory for Endocrine Tumor, Shanghai National Center for Translational Medicine, Ruijin Hospital, Shanghai Jiao Tong University School of Medicine, Shanghai 200025, China; Department of Endocrine and Metabolic Diseases, The First Affiliated Hospital of Guangxi Medical University, Nanning, Guangxi 530021, China; Department of Endocrine and Metabolic Diseases, The First Hospital of Lanzhou University, Lanzhou, Gansu 730000, China; Zhejiang Provincial Center for Disease Control and Prevention, Hangzhou, Zhejiang 310051, China; Zhejiang Provincial Center for Disease Control and Prevention, Hangzhou, Zhejiang 310051, China; Department of Endocrine and Metabolic Diseases, The Affiliated Hospital of Guiyang Medical College, Guiyang, Guizhou 550004, China; Department of Endocrine and Metabolic Diseases, Xinhua Hospital Affiliated to Shanghai Jiao Tong University School of Medicine, Shanghai 200092, China; Department of Endocrine and Metabolic Diseases, Tongji Hospital, Tongji Medical College, Huazhong University of Science and Technology, Wuhan, Hubei 430022, China; Department of Endocrine and Metabolic Diseases, Sun Yat-sen Memorial Hospital, Sun Yat-sen University, Guangzhou, Guangdong 510120, China; Department of Endocrine and Metabolic Diseases, The Affiliated Hospital of Southwest Medical University, Luzhou, Sichuan 646000, China; Department of Endocrine and Metabolic Diseases, Fujian Provincial Hospital, Fujian Medical University, Fuzhou, Fujian 350003, China; Department of Endocrine and Metabolic Diseases, Central Hospital of Dalian University of Technology, Dalian, Liaoning 116033, China; Department of Endocrine and Metabolic Diseases, The First Hospital of Jilin University, Changchun, Jilin 130021, China; Department of Endocrine and Metabolic Diseases, The First Affiliated Hospital of Wenzhou Medical University, Wenzhou, Zhejiang 325000, China; Department of Endocrine and Metabolic Diseases, The First Affiliated Hospital of Wenzhou Medical University, Wenzhou, Zhejiang 325000, China; Department of Endocrine and Metabolic Diseases, The First Affiliated Hospital of Guangxi Medical University, Nanning, Guangxi 530021, China; Department of Endocrine and Metabolic Diseases, Qilu Hospital of Shandong University, Jinan, Shandong 250012, China; Department of Endocrine and Metabolic Diseases, Qilu Hospital of Shandong University, Jinan, Shandong 250012, China; Department of Endocrine and Metabolic Diseases, Jiangxi Provincial People’s Hospital Affiliated to Nanchang University, Nanchang, Jiangxi 330006, China; Department of Endocrine and Metabolic Diseases, The Second Affiliated Hospital of Harbin Medical University, Harbin, Heilongjiang 150086, China; Department of Endocrine and Metabolic Diseases, Central Hospital of Shanghai Jiading District, Shanghai 201800, China; Department of Endocrine and Metabolic Diseases, Union Hospital, Tongji Medical College, Huazhong University of Science and Technology, Wuhan, Hubei 430022, China; Department of Endocrine and Metabolic Diseases, Jiangsu Province Hospital on Integration of Chinese and Western Medicine, Nanjing, Jiangsu 210028, China; Department of Endocrine and Metabolic Diseases, The First Affiliated Hospital of Anhui Medical University, Hefei, Anhui 230022, China; Department of Endocrine and Metabolic Diseases, The First Affiliated Hospital of Nanjing Medical University, Nanjing, Jiangsu 210029, China; Department of Endocrine and Metabolic Diseases, The First Affiliated Hospital of Chongqing Medical University, Chongqing 400016, China; Department of Endocrine and Metabolic Diseases, Union Hospital, Tongji Medical College, Huazhong University of Science and Technology, Wuhan, Hubei 430022, China; Department of Endocrine and Metabolic Diseases, Shandong Provincial Hospital affiliated to Shandong University, Jinan, Shandong 250021, China; Department of Endocrine and Metabolic Diseases, Chinese People’s Liberation Army General Hospital, Beijing 100853, China; Department of Endocrine and Metabolic Diseases, Shanghai Institute of Endocrine and Metabolic Diseases, Ruijin Hospital, Shanghai Jiao Tong University School of Medicine, Shanghai 200025, China; Shanghai National Clinical Research Center for Metabolic Diseases, Key Laboratory for Endocrine and Metabolic Diseases of the National Health Commission of the P.R. China, Shanghai Key Laboratory for Endocrine Tumor, Shanghai National Center for Translational Medicine, Ruijin Hospital, Shanghai Jiao Tong University School of Medicine, Shanghai 200025, China; Department of Endocrine and Metabolic Diseases, Shanghai Institute of Endocrine and Metabolic Diseases, Ruijin Hospital, Shanghai Jiao Tong University School of Medicine, Shanghai 200025, China; Shanghai National Clinical Research Center for Metabolic Diseases, Key Laboratory for Endocrine and Metabolic Diseases of the National Health Commission of the P.R. China, Shanghai Key Laboratory for Endocrine Tumor, Shanghai National Center for Translational Medicine, Ruijin Hospital, Shanghai Jiao Tong University School of Medicine, Shanghai 200025, China; Department of Endocrine and Metabolic Diseases, Shanghai Institute of Endocrine and Metabolic Diseases, Ruijin Hospital, Shanghai Jiao Tong University School of Medicine, Shanghai 200025, China; Shanghai National Clinical Research Center for Metabolic Diseases, Key Laboratory for Endocrine and Metabolic Diseases of the National Health Commission of the P.R. China, Shanghai Key Laboratory for Endocrine Tumor, Shanghai National Center for Translational Medicine, Ruijin Hospital, Shanghai Jiao Tong University School of Medicine, Shanghai 200025, China; Department of Endocrine and Metabolic Diseases, Shanghai Institute of Endocrine and Metabolic Diseases, Ruijin Hospital, Shanghai Jiao Tong University School of Medicine, Shanghai 200025, China; Shanghai National Clinical Research Center for Metabolic Diseases, Key Laboratory for Endocrine and Metabolic Diseases of the National Health Commission of the P.R. China, Shanghai Key Laboratory for Endocrine Tumor, Shanghai National Center for Translational Medicine, Ruijin Hospital, Shanghai Jiao Tong University School of Medicine, Shanghai 200025, China; Department of Endocrine and Metabolic Diseases, Shanghai Institute of Endocrine and Metabolic Diseases, Ruijin Hospital, Shanghai Jiao Tong University School of Medicine, Shanghai 200025, China; Shanghai National Clinical Research Center for Metabolic Diseases, Key Laboratory for Endocrine and Metabolic Diseases of the National Health Commission of the P.R. China, Shanghai Key Laboratory for Endocrine Tumor, Shanghai National Center for Translational Medicine, Ruijin Hospital, Shanghai Jiao Tong University School of Medicine, Shanghai 200025, China

**Keywords:** cardiovascular disease, diabetes, hemoglobin, systolic blood pressure, LDL-C

## Abstract

Optimal control of hemoglobin A1c (HbA1c), blood pressure, and cholesterol (ABC risk factors) is essential for reducing cardiovascular disease (CVD) risk in individuals with diabetes. However, age-specific contributions of these factors remain inadequately characterized. Using data from the China Cardiometabolic Disease and Cancer Cohort study, we assessed the associations between ABC risk factors and incident CVD among Chinese adults with diabetes, stratified by age groups of < 55, 55 to < 65, 65 to < 75, and ≥ 75 years. Cox proportional hazards models and population-attributable fractions (PAFs) were used to quantify the associations between ABC risk factors and incident CVD. During a median follow-up of 10.1 years, 4707 incident cases of CVD were documented. Higher levels of baseline HbA1c, systolic blood pressure (SBP), and low-density lipoprotein cholesterol (LDL-C) were significantly associated with increased CVD risk. Age modified these associations (*P*_interaction_ < 0.05), with progressively attenuated hazard ratios (HRs) observed in older age groups. Compared with HbA1c < 7.0%, HbA1c ≥ 9.0% showed stronger CVD associations in adults aged < 55 years (HR = 2.42; 95% confidence interval [CI]: 1.98–2.97) than in those aged ≥ 75 years (HR = 1.50; 95% CI: 1.12–2.02), and SBP ≥ 140 mmHg and LDL-C ≥  4.1 mmol/L were significant only in younger groups. The leading contributor to PAFs for CVD was SBP (28.3%), followed by HbA1c (12.0%) and LDL-C (9.2%), with diminishing impacts across older groups. These results underscore the importance of age-specific management of ABC risk factors in diabetes care, with the benefit of stricter risk factor management in younger adults and the need for a more flexible approach in older populations.

## Introduction

Diabetes is a highly prevalent chronic condition and a major contributor to the global burden of disease, imposing substantial challenges on healthcare systems [1]. China has experienced a continuous rise in diabetes prevalence and bears the highest diabetes burden [2, 3]. Cardiovascular disease (CVD) remains the leading complication of diabetes. Patients with diabetes have a two- to four-fold increased risk of cardiovascular morbidity and mortality compared to the general population [4–7].

Management of key modifiable variables, including glycated hemoglobin A1c (HbA1c), blood pressure (BP), and low-density lipoprotein cholesterol (LDL-C), collectively termed ABC risk factors, has been consistently associated with reduced cardiovascular risk in patients with type 2 diabetes [8–13]. Multifactorial risk factor management, as demonstrated in studies such as the Steno-2 trial, has been shown to significantly reduce cardiovascular morbidity and mortality in patients with diabetes [14]. Evidence from the Swedish nationwide registry study also suggests that optimal control of key risk factors could eliminate the excess risk of myocardial infarction in individuals with diabetes [5].

However, emerging evidence suggests that the benefits of managing metabolic risk factors may vary across age groups [15, 16]. Abdel-Rahman *et al*. [9] reported that achieving target levels of ABC risk factors is associated with reduced cardiac morbidity. Furthermore, age appears to modify these associations, with stronger effects observed in individuals aged < 65 years. Similarly, Rawshani *et al*. [5] identified a monotonic increase in CVD risk with a greater number of risk factors outside target ranges among younger patients, suggesting greater potential gains from aggressive management in this population. In contrast, studies among older adults (≥ 75 years) revealed paradoxical associations, indicating that achieving strict HbA1c levels (6.5%–6.9%) was associated with a higher risk of ischemic heart disease compared with a more relaxed glycemic target (≥ 7.5%) [17]. Collectively, these findings underscore the need for a deeper understanding of age-related variations in the effectiveness of ABC risk factors on cardiovascular outcomes, particularly in the context of deve­loping countries experiencing a surge in diabetes, such as China, where age-specific evidence remains very limited.

To address this gap, we analyzed data from the China Cardiometabolic Disease and Cancer Cohort (4C), a nationwide 10-year prospective study, to evaluate age-dependent associations between ABC status and CVD risk among Chinese adults with diabetes. Our study aimed to explore age-related differences in ABC status and their associations with cardiovascular outcomes in individuals with diabetes, supporting considerations for age-specific diabetes management.

## Results

### Participant characteristics

**Figure 1:**
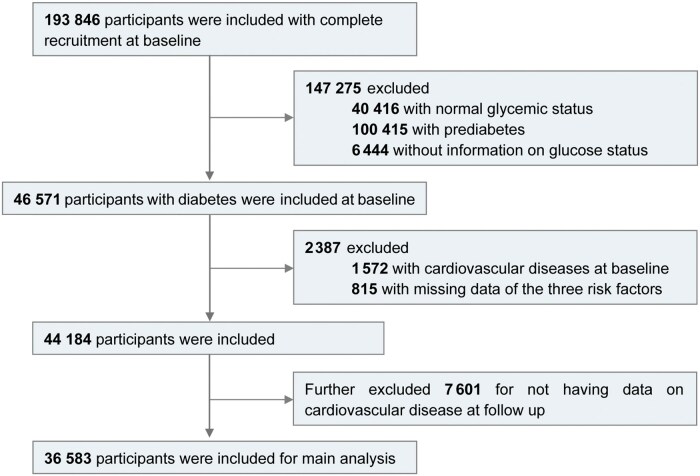
Participant flow diagram of the China Cardiometabolic Disease and Cancer Cohort (4C) Study.

A total of 193 846 participants aged 40 years or older were enrolled from 20 communities across various geographic regions of the Chinese mainland between January 2011 and December 2012 [6, 13, 18, 19]. At baseline, 46 571 participants were diagnosed with diabetes. Participants were followed up until 30 November 2021, with a median follow-up duration of 10.1 years. After excluding 1572 participants with pre-existing CVD, 815 with missing data for baseline HbA1c, systolic blood pressure (SBP), or LDL-C, and 7601 with missing follow-up CVD data due to incomplete disease surveillance coverage in certain regions, a total of 36 583 participants were included in the final main analysis (Fig. 1; Supplementary Table S1).

The mean age of participants was 60.04 years (standard deviation [SD] 8.87), and men accounted for 38.1% of the study population. Baseline characteristics stratified by age groups are presented in Table 1. Overall, 28.4% of participants were younger than 55 years, 42.3% were aged 55 to < 65 years, 24.8% were 65 to < 75 years, and 4.4% were 75 years or older (Supplementary Table S2). Compared with the young group, older individuals were more likely to be women, had higher levels of SBP and LDL-C, and were more frequently taking antihypertensive, lipid-lowering, and glucose-lowering medications. Conversely, they had lower body mass index (BMI), fasting plasma glucose (FPG), HbA1c, and triglycerides, as well as lower proportions of current smokers and drinkers (*P *< 0.001).

**Table 1:** Baseline clinical characteristics of the study participants by age groups.^a^

Characteristic	Total (*n *= 36 583)	Age group				*P* value^*^
		Young (< 55 years; *n *= 10 399)	Middle-aged (55 to < 65 years; *n *= 15 476)	Old (65 to < 75 years; *n *= 9084)	Elderly (≥ 75 years; *n *= 1624)	
**Age, year**	60.04 ± 8.87	49.36 ± 4.10	59.90 ± 2.80	69.24 ± 2.80	78.25 ± 3.11	< 0.001
**Male, *n* (%)**	13 941 (38.1%)	4275 (41.1%)	5649 (36.5%)	3337 (36.7%)	680 (41.9%)	< 0.001
**Current smoking, *n* (%)**	5082 (14.6%)	1991 (20.2%)	2130 (14.5%)	843 (9.7%)	118 (7.6%)	< 0.001
**Current drinking, *n* (%)**	3711 (10.8%)	1380 (14.2%)	1574 (10.9%)	649 (7.6%)	108 (7.0%)	< 0.001
**High school or further education, *n* (%)**	11 306 (31.8%)	4223 (42.0%)	4023 (26.8%)	2723 (30.7%)	337 (21.2%)	< 0.001
**FPG (mmol/L)**	7.78 ± 2.59	8.05 ± 2.89	7.76 ± 2.55	7.56 ± 2.29	7.47 ± 2.38	< 0.001
**2 h-PG (mmol/L)**	13.32 ± 4.94	13.20 ± 5.12	13.33 ± 4.96	13.40 ± 4.73	13.51 ± 4.73	0.001
**HbA1c (%)**	7.15 ± 1.57	7.19 ± 1.72	7.16 ± 1.55	7.10 ± 1.42	7.13 ± 1.50	< 0.001
**SBP (mmHg)**	140.41 ± 21.31	134.11 ± 20.08	141.04 ± 21.06	145.02 ± 21.05	148.91 ± 22.26	< 0.001
**DBP (mmHg)**	80.50 ± 11.37	82.32 ± 11.62	81.24 ± 10.99	78.07 ± 11.10	75.41 ± 11.08	< 0.001
**LDL-C (mmol/L)**	2.96 ± 0.91	2.91 ± 0.91	2.98 ± 0.91	2.97 ± 0.92	3.02 ± 0.96	<0.001
**HDL-C (mmol/L)**	1.27 ± 0.34	1.26 ± 0.35	1.27 ± 0.34	1.27 ± 0.35	1.28 ± 0.34	0.004
**TC (mmol/L)**	5.12 ± 1.21	5.07 ± 1.23	5.15 ± 1.20	5.11 ± 1.21	5.14 ± 1.24	0.016
**Triglycerides (mmol/L)**	1.61 (1.12–2.37)	1.65 (1.12–2.51)	1.63 (1.13–2.40)	1.55 (1.10–2.22)	1.49 (1.06–2.07)	< 0.001
**BMI (kg/m^2^)**	25.74 ± 3.77	25.9 ± 3.75	25.78 ± 3.72	25.57 ± 3.82	25.38 ± 3.94	< 0.001
**Anti-hypertensive medication, *n* (%)**	6415 (17.5%)	1103 (10.6%)	2814 (18.2%)	2138 (23.5%)	360 (22.2%)	< 0.001
**Lipid-lowering medication, *n* (%)**	428 (1.2%)	69 (0.7%)	204 (1.3%)	134 (1.5%)	21 (1.3%)	< 0.001
**Glucose-lowering medication, *n* (%)**	11 457 (31.3%)	2669 (25.7%)	4911 (31.7%)	3306 (36.4%)	571 (35.2%)	< 0.001

a Values are mean (SD) or *n* (%).

* *P* values are based on one-way analysis of variance for continuous variables or Chi-square test for categorical variables.

Abbreviations: 2 h-PG, 2-h post-load glucose; BMI, body mass index; DBP, diastolic blood pressure; FPG, fasting plasma glucose; HDL-C, high-density lipoprotein cholesterol; HbA1c, Hemoglobin A1c; LDL-C, low-density lipoprotein cholesterol; SBP, systolic blood pressure; TC, total cholesterol.

### ABC levels by age groups

**Figure 2:**
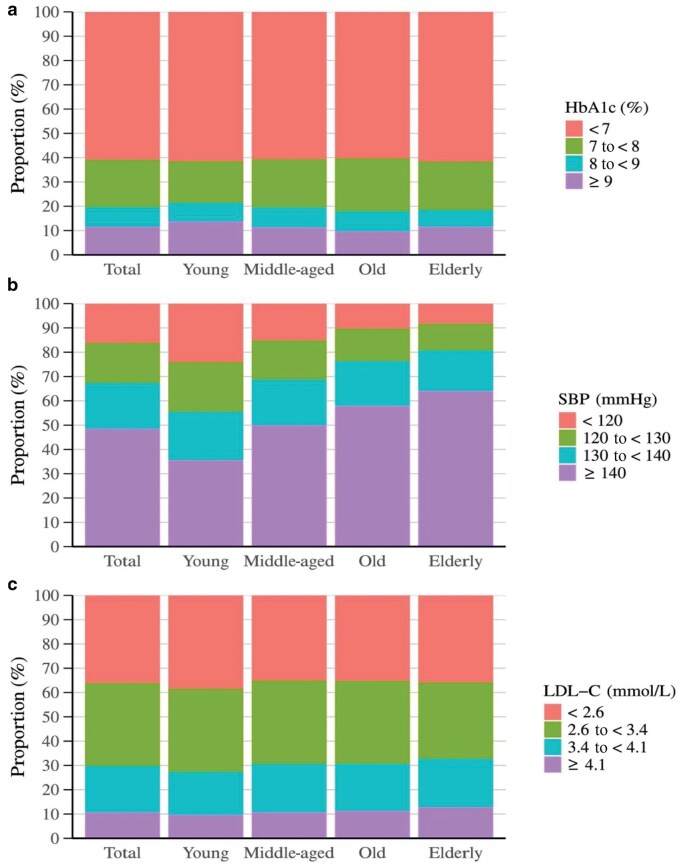
Distribution of (a) HbA1c, (b) SBP, and (c) LDL-C across age strata in Chinese adults with diabetes.

Approximately two-thirds of patients with diabetes had HbA1c levels < 7%, while nearly one-third had SBP < 130 mmHg or LDL-C <  2.6 mmol/L (Fig. 2). The proportion of individuals with HbA1c < 7% was the highest in the elderly group (≥ 75 years). However, the proportions of individuals achieving SBP < 130 mmHg and LDL-C <  2.6 mmol/L decreased progressively with increasing age.

### Association of ABC risk factors with CVD by age groups

**Figure 3:**
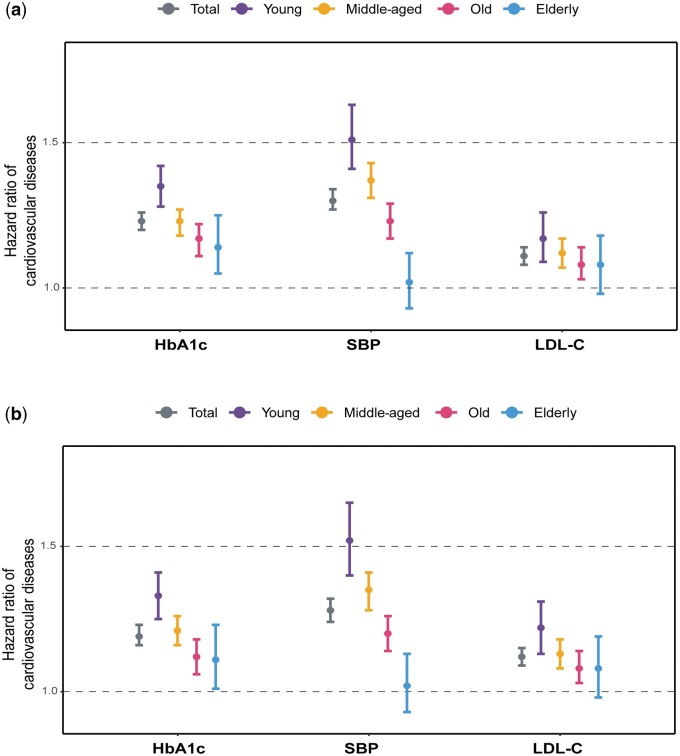
Associations of per SD increase in ABC risk factors with incident CVD across age strata. (a) Model 1 is adjusted for age and sex. (b) Model 2 is further adjusted for body-mass index, current smoking, current drinking, educational attainment, and receiving glucose-lowering medication, lipid-lowering medication, and anti-hypertensive medication at baseline.

During a median follow-up of 10.1 years, 4707 cases of CVD events occurred. The associations between each risk factor (HbA1c, SBP, and LDL-C; collectively referred to as ABC risk factors) and incident CVD risk, stratified by age categories, are presented in Table 2. Overall, higher levels of HbA1c, SBP, and LDL-C were associated with increased risks of CVD (Table 2; Fig. 3). For example, compared with HbA1c < 7.0%, the adjusted hazard ratios (HRs) (95% confidence intervals [CIs]) for HbA1c levels of 7% to < 8%, 8% to < 9%, and ≥ 9% were 1.14 (1.05–1.23), 1.39 (1.25–1.54), and 1.73 (1.58–1.89), respectively (Table 2).

**Table 2:** Associations of ABC risk factors with incident CVD across age strata.^a^

		Age group				*P_interaction_*
	Total (*n *= 36 583)	Young (< 55 years; *n *= 10 399)	Middle-aged (55 to < 65 years; *n *= 15 476)	Old (65 to < 75 years; *n *= 9084)	Elderly (≥ 75 years; *n *= 1624)	
**HbA1c (%)**						
** < 7**	1.00 (Ref)	1.00 (Ref)	1.00 (Ref)	1.00 (Ref)	1.00 (Ref)	< 0.001
** 7 to < 8**	1.14 (1.05–1.23)	1.02 (0.80–1.30)	1.26 (1.11–1.43)	1.07 (0.94–1.22)	1.04 (0.79–1.36)	
** 8 to < 9**	1.39 (1.25–1.54)	1.39 (1.03–1.86)	1.56 (1.32–1.83)	1.27 (1.07–1.52)	1.19 (0.81–1.74)	
**≥ 9**	1.73 (1.58–1.89)	2.42 (1.98–2.97)	1.79 (1.56–2.06)	1.43 (1.22–1.68)	1.50 (1.12–2.02)	
**SBP (mmHg)**						
** < 120**	1.00 (Ref)	1.00 (Ref)	1.00 (Ref)	1.00 (Ref)	1.00 (Ref)	< 0.001
** 120 to < 130**	1.19 (1.05–1.35)	1.38 (1.03–1.86)	1.14 (0.94–1.38)	1.07 (0.85–1.35)	1.26 (0.79–2.03)	
** 130 to < 140**	1.21 (1.07–1.37)	1.51 (1.13–2.02)	1.10 (0.91–1.33)	1.11 (0.89–1.37)	1.21 (0.78–1.88)	
** ≥ 140**	1.71 (1.54–1.91)	2.41 (1.87–3.10)	1.71 (1.46–2.01)	1.49 (1.23–1.80)	1.22 (0.82–1.81)	
**LDL-C (mmol/L)**						
** < 2.6**	1.00 (Ref)	1.00 (Ref)	1.00 (Ref)	1.00 (Ref)	1.00 (Ref)	0.013
** 2.6 to < 3.4**	1.10 (1.02–1.18)	1.29 (1.06–1.57)	1.04 (0.93–1.17)	1.07 (0.95–1.20)	1.24 (0.97–1.58)	
** 3.4 to < 4.1**	1.28 (1.18–1.39)	1.38 (1.09–1.74)	1.28 (1.12–1.46)	1.25 (1.08–1.43)	1.30 (0.99–1.71)	
** ≥ 4.1**	1.33 (1.20–1.47)	1.82 (1.39–2.37)	1.40 (1.19–1.65)	1.16 (0.98–1.38)	1.15 (0.82–1.61)	

a Adjusted for age, sex, body-mass index, current smoking, current drinking, educational attainment, and receiving glucose-lowering medication, lipid-lowering medication, and anti-hypertensive medication at baseline. *P* values for the interaction between age group and each individual risk factor are shown to evaluate variations in the associations between individual risk factors and incident CVD across different age groups.

Significant interactions were observed between ABC risk factors and age groups, indicating that HRs varied significantly across age categories. In age-stratified models, HbA1c ≥ 9% was associated with an increased CVD risk across all age groups. The strongest association was observed in the young group (HR = 2.42, 95% CI: 1.98–2.97), followed by the middle-aged group (HR = 1.79, 95% CI: 1.56–2.06). The associations were weaker in the old and elderly groups, with HRs of 1.43 (1.22–1.68) and 1.50 (1.12–2.02), respectively. For SBP ≥ 140 mmHg, the young group exhibited the strongest association with CVD risk (HR = 2.41, 95% CI: 1.87–3.10). Although the risk increased with SBP elevation in the middle-aged and old groups, the HRs were smaller than those in the young group (*P*_interaction_ < 0.001). No significant association was observed in the elderly group (HR = 1.22, 95% CI: 0.82–1.81). A similar trend was noted for LDL-C ≥ 4.1 mmol/L, with the strongest association observed in the young group (HR = 1.82, 95% CI: 1.39–2.37), and no significant association in the elderly group (HR = 1.15, 95% CI: 0.82–1.61; Table 2). Furthermore, as shown in Fig. 3, the CVD risk per SD increase in HbA1c, SBP, and LDL-C decreased with advancing age, with the highest HRs per SD observed in the young group.

The findings presented in Table 2 indicate that the exposure levels at which HbA1c, SBP, and LDL-C become significantly associated with CVD risk differed by age group. For HbA1c, younger participants showed increased CVD risk at lower exposure levels (> 7% for the middle-aged group, > 8% for the old group, and ≥ 9% for the elderly group). For SBP, even levels ≥ 120 mmHg were associated with increased risk in the young group, whereas a higher exposure level (≥ 140 mmHg) was needed for the old group. For LDL-C, a level of ≥ 2.6 mmol/L was associated with an increased risk in the young group, and ≥ 3.4 mmol/L in the middle-aged group. Notably, among the old group, only LDL-C levels between 3.4 and 4.1 mmol/L were significantly associated with increased CVD risk (HR = 1.25, 95% CI: 1.08–1.43), whereas no significant association was observed for LDL-C ≥ 4.1 mmol/L (HR = 1.16, 95% CI: 0.98–1.38), compared with LDL-C < 2.6 mmol/L (Table 2). Using restricted cubic splines, the continuous associations of HbA1c, SBP, and LDL-C with CVD risk indicated patterns generally consistent with those observed in the categorical analyses (Supplementary Fig. S1).

Sensitivity analyses among individuals receiving glucose-lowering medications revealed a similar trend to the primary analysis (Supplementary Table S3). Models conducted without adjusting for age within age-stratified analyses and using Fine–Gray competing-risks analyses produced comparable results (Supplementary Tables S4 and S5). After excluding participants with cancer or reduced renal function (estimated glomerular filtration rate [eGFR] < 60 mL/min/1.73 m^2^) at baseline, and after further adjusting for baseline comorbidities, renal function, and healthcare access, the findings remained consistent with the main analysis (Supplementary Tables S6 and S7).

### PAFs for CVD associated with ABC risk factors

**Figure 4:**
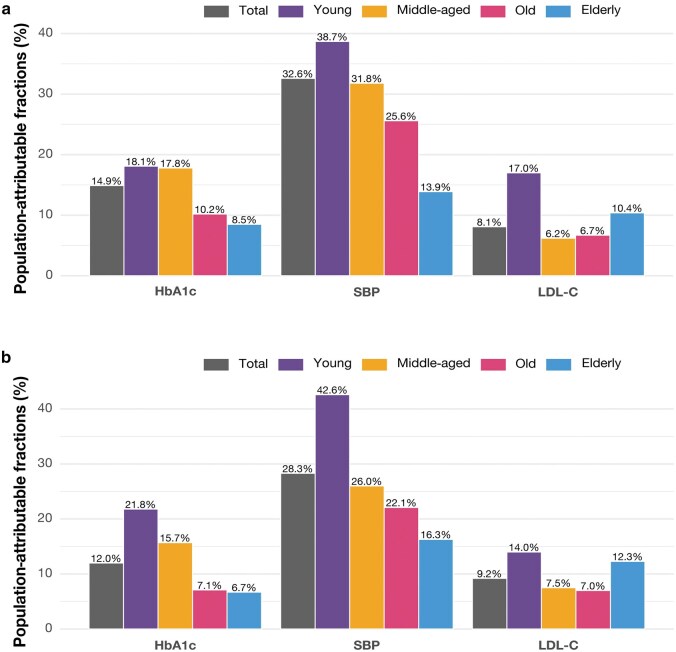
PAFs for CVD associated with ABC risk factors across different age groups. (a) Model 1 is adjusted for age and sex. (b) Model 2 is further adjusted for body mass index, current smoking, current drinking, educational attainment, receiving glucose-lowering medication, lipid-lowering medication, and anti-hypertensive medication at baseline.

Figure 4 illustrates the proportions of CVD incidence attributable to ABC risk factors across age groups. In total, SBP had the highest PAFs at 28.3%, followed by HbA1c (12.0%) and LDL-C (9.2%). Across all age groups, SBP exhibited the highest PAFs for CVD risk, though its impact declined with advancing age. The highest PAF was observed in the young group (42.6%), followed by the middle-aged (26.0%), old (22.1%), and elderly (16.3%) groups. Similarly, elevated HbA1c contributed substantially to CVD incidence, with the highest PAF in the young group (21.8%), decreasing to 15.7% in the middle-aged group. The PAFs for HbA1c were lower in the old (7.1%) and elderly (6.7%) groups, suggesting a decreasing impact with increasing age. The overall impact of LDL-C on CVD risk was modest. The highest PAF was observed in the young group (14.0%), while the lowest was in the old group (7.0%). Notably, the PAF for LDL-C in the elderly group (12.3%) was higher compared to the middle-aged (7.5%) and old groups (7.0%).

## Discussion

In this 10-year prospective cohort study of 36 583 adults with diabetes in China, we demonstrated clear age-related heterogeneity in the associations of glucose, blood pressure, and lipid levels with CVD risk. Although higher levels of HbA1c, SBP, and LDL-C were each associated with increased CVD risk, the strength of these associations attenuated with advancing age. Notably, the levels of these factors associated with excess CVD risk were generally higher in older adults. Among individuals aged ≥ 75 years, an increased CVD risk was observed only in higher HbA1c categories (≥ 9%), whereas elevated SBP and LDL-C levels were not significantly associated with CVD outcomes. These findings suggest that the cardiovascular relevance of traditional ABC risk factors diminishes with aging, highlighting substantial heterogeneity in risk factor–disease relationships across the life course.

Previous studies have demonstrated that elevated levels of HbA1c, SBP, and LDL-C independently contribute to increased CVD risk [9, 20–22]. Our analysis further revealed that younger individuals with diabetes exhibited a higher CVD risk associated with elevated ABC risk factors, whereas these associations weakened progressively in older populations. This trend is consistent with findings from an Israel cohort, which observed that achieving effective levels of ABC risk factors was associated with reduced cardiac morbidity in a dose-dependent manner, with stronger associations among individuals aged < 65 years [9]. However, our study provides a more detailed age stratification, revealing further distinctions, particularly between the old group (65 to < 75 years) and the elderly group (≥ 75 years). Individuals aged ≥ 75 years are often considered physiologically and metabolically distinct from the average middle-aged or younger adults with diabetes. Wan *et al*. [20] reported a stronger association between HbA1c and CVD risk in individuals aged 45–54 years than in those aged 75–84 years. Regarding SBP, elevated levels are associated with increased cardiovascular risk in younger and middle-aged adults but not consistently among older populations, with some studies reporting increased risk at substantially higher SBP levels (e.g. > 154 mmHg) [23, 24]. Consistently, our study demonstrated that in individuals aged ≥ 75 years, even SBP ≥ 140 mmHg was not significantly associated with increased CVD risk (HR = 1.22, 95% CI: 0.82–1.81). These findings support the notion that younger patients require more intensive management of ABC risk factors, whereas treatment targets may be appropriately adjusted in older individuals. In particular, for those aged ≥ 75 years, a more individualized management approach is needed.

Additionally, our PAF analysis further supports this age-dependent pattern. The potential reduction in CVD events attributable to these factors diminished with age, suggesting that interventions targeting ABC risk factors may yield greater benefits in younger populations. However, the PAF estimates in our study should be interpreted with caution. PAF calculation assumes that risk factors exert independent and causal effects on outcomes. In reality, the ABC factors, such as HbA1c and SBP, are interrelated and may interact, which could result in double-counting or biased estimates when considered jointly. Therefore, the PAF values presented here should be regarded as hypothetical indicators of the potential proportional reduction in CVD risk under the situation that each factor is independently controlled to its target level within our study population, rather than as precise additive contributions. Despite this limitation, effective management of ABC risk factors remains crucial in older adults. While other determinants may play roles in late-life cardiovascular risk, tailored interventions for older individuals may still contribute to the reduction of overall CVD burden.

The attenuated associations between ABC risk factors and CVD risk in older adults may be partly explained by age-related vascular adaptations and differential metabolic responses [25, 26]. Declining β-cell function and increased insulin resistance with aging reduce glycemic control capacity [25]. HbA1c may no longer accurately capture modifiable glycemic burden or short-term glucose fluctuations that contribute to vascular injury, thereby potentially diminishing its predictive value for CVD [27]. Vascular aging, characterized by arterial stiffening, medial calcification, and reduced endothelial compliance, further attenuates the relationship between blood pressure fluctuations and endothelial injury [28]. In addition, advanced arterial remodeling and lipid accumulation in older adults may diminish the incremental pathogenic impact of elevated LDL-C [26, 29]. These vascular alterations in older adults may diminish the direct impact of SBP and LDL-C on CVD risk [30]. Therefore, younger individuals may be more sensitive to changes in these risk factors. Moreover, the presence of multimorbidity in older adults, including chronic kidney disease and malignancies, may overshadow the contributions of traditional risk factors [31]. Notably, while the absolute impact of LDL-C on CVD risk diminished with age, its PAF increased in individuals aged ≥ 75 years, likely due to the relatively reduced contributions of HbA1c and SBP.

These findings through age-stratified analyses support the importance of management strategies that incorporate age-specific targets for glucose, blood pressure, and lipid levels. Among older adults, a more flexible approach to risk factor management may be appropriate, considering the risk-benefit balance. Evidence suggests that overly strict targets, particularly for HbA1c and SBP, may increase the risk of adverse events such as hypoglycemia, cognitive decline, and hemorrhagic stroke [32]. Similarly, excessively low LDL-C levels in older adults may be associated with frailty, neurodegenerative diseases, and impaired immune function [33, 34]. These observations support the importance of individualized, age-specific risk factor management strategies, particularly in older populations where clinicians should carefully weigh potential risks against benefits rather than focus solely on treatment effectiveness.

In conclusion, our study provided population-based evidence supporting age-specific management of glycemia, blood pressure, and lipids in individuals with diabetes. Stronger associations and lower risk thresholds in younger adults suggest greater potential benefit from intensive risk factor control earlier in life, whereas attenuated associations in older populations, particularly among those aged 75 years or older, underscore the need to balance cardiovascular risk reduction against treatment burden and competing risks. These results support an age-adapted approach to ABC risk factor management that incorporates age and overall clinical context to optimize cardiovascular outcomes in diabetes care.

## Limitations of the study

First, although we adjusted for multiple confounders, the possibility of residual confounding remains, such as diabetes duration and other unmeasured or inadequately measured factors that may have influenced the observed associations. Second, the study population primarily consisted of middle-aged and older Chinese adults, which may limit the generalizability of our findings to other populations, such as younger individuals, other race/ethnic groups, and patients with other specific diseases or conditions. Moreover, the number of participants aged ≥ 75 years was relatively small, potentially influencing the statistical power to detect modest associations in this age group. Third, our study relied on a single baseline measurement of ABC risk factors, which overlooked intra-individual variation and changes in treatment over time. This could introduce misclassification in exposure classification and regression dilution, leading to an underestimation of the true associations [35, 36], especially among older adults whose metabolic profiles tend to fluctuate more over time. Future studies incorporating repeated measurements are warranted to clarify the impact of dynamic changes in ABC control on cardiovascular outcomes. In addition, cardiovascular outcomes were analyzed as a compo­site endpoint, and the absence of subtype-specific cardiovascular analyses may have obscured potential heterogeneity across diffe­rent CVD phenotypes. Finally, survival bias may also be present, as individuals who reach older age may have better cardiovascular resilience or lower susceptibility to CVD, or they may have already modified certain risk factors through lifestyle changes.

## Materials and methods

### Study design and population

The 4C Study was a prospective, multicenter, population-based cohort study designed to explore risk factors for diabetes, CVDs, and all-cause mortality in the Chinese population. The study design has been previously described in detail [6, 13]. In brief, a total of 20 communities from various geographic regions were selected to represent the general population in mainland China. There was no restriction on gender or ethnicity. Each eligible subject was approached by trained local community workers using a door-to-door invitation method. Overall, 193 846 participants aged 40 years or older underwent a baseline examination and were enrolled in the 4C study between January 2011 and December 2012. Among them, 46 571 participants were diagnosed with diabetes at baseline.

This study was approved by the Medical Ethics Committee of Ruijin Hospital, Shanghai Jiao Tong University. All study participants provided written informed consent.

### Baseline data collection

Baseline data were collected at local community clinics by trained personnel following a standardized protocol. Participants completed a standard questionnaire capturing sociodemographic information, lifestyle risk factors, and medical history, including medication use for glucose-lowering, lipid-lowering, and blood pressure management. Current smokers were defined as indivi­duals who smoked at least seven cigarettes per week for 6 months or more. Current drinkers were defined as those who consumed alcohol at least once weekly in the past 6 months. Educational attainment was classified as less than high school and high school or above.

Height and weight were measure using standard methods, and BMI was calculated as weight (kilogram) divided by the square of height (square meters). Blood pressure was measured three times using an automated electronic device (OMRON Model HEM-752 FUZZY) after at least 5 min of seated rest, with the average of the three measurements used for analysis.

All participants underwent an oral glucose tolerance test after fasting for at least 10 h, with blood samples collected at 0 and 2 h. Plasma glucose concentrations were measured locally using the glucose oxidase or hexokinase method. HbA1c was analyzed within 4 weeks of collection using high-performance liquid chromatography (VARIANT II Hemoglobin Testing System, Bio-Rad Laboratories). Lipid profiles, including LDL-C, were assessed at the central laboratory using an automated analyzer (ARCHITECT ci16200).

### Outcome assessment

Cardiovascular events during follow-up were obtained through participant identification numbers linked to the National Disease Surveillance Point System, Cardiovascular Disease Registries, and the National Health Insurance System. Diagnoses were coded according to the International Classification of Diseases, 10th Revision (ICD-10). Incident CVD was defined as the first occurrence of myocardial infarction, stroke, or cardiovascular death. The specific ICD-10 codes used to define CVD events are provided in Supplementary Table S8.

### Statistical analyses

Participants diagnosed with diabetes at baseline were categorized into four age groups: young (< 55 years), middle-aged (55 to < 65 years), old (65 to < 75 years), and elderly (≥ 75 years) groups [37, 38]. Baseline characteristics were summarized by age groups, with continuous variables presented as means (SDs) or medians (interquartile ranges [IQRs]) and categorical variables as numbers (percentages).

HbA1c levels were categorized into four categories: < 7%, 7% to < 8%, 8% to < 9%, and ≥ 9%; SBP was stratified into < 120, 120 to < 130, 130 to < 140, and ≥ 140 mmHg; and LDL-C levels were grouped as < 2.6 mmol/L, 2.6 to < 3.4 mmol/L, 3.4 to < 4.1 mmol/L, and ≥ 4.1 mmol/L, based on guideline-recommended targets and prior epidemiological studies [39–42].

Cox proportional hazards regression models were used to examine the associations between baseline ABC risk factors (including HbA1c, SBP, and LDL-C) and the risks of incident CVD across age groups. HRs and 95% CIs were calculated. Models were adjusted for age, sex, BMI, current smoking, current drinking, educational attainment, use of glucose-lowering medication, lipid-lowering medication, and antihypertensive medication. Because missing data in the current study was minimal (approximately 1%–2% for all covariates), we performed complete-case analyses. All models were constructed using the available data without imputation. To assess differences in the associations across age groups, interaction terms (age group × each risk factor) were incorporated into the models, and *P*-values for interactions were calculated. Several sensitivity analyses were performed to assess the robustness of the findings: (i) analyses restricted to participants with diabetes receiving glucose-lowering medications; (ii) analyses without age adjustment within the age-stratified models to evaluate potential residual confounding by intra-stratum age variation; (iii) competing-risk analyses using Fine–Gray subdistribution hazard models to account for non-CVD death; (iv) exclusion of participants with a history of cancer and those with reduced renal function (eGFR < 60 mL/min/1.73 m^2^) at baseline; and (v) models further adjusted for baseline presence of comorbidities, renal function (eGFR), and healthcare access (including the number of hospital beds, physicians, and density of each center).

To estimate population-level burden of CVD attributable to these risk factors, we calculated the PAFs and 95% CIs for each ABC risk factor using the methodological framework proposed by Ferguson and Connell [43]. Consistent with the HR estimation, PAFs were calculated based on the aforementioned multivariable-adjusted models. The results were visualized as bar plots to illustrate the contributions of each risk factor to CVD incidence in the total and age-specific populations.

All analyses were conducted using SAS version 9.2 and R (version 4.4.1), and a two-tailed *P *< 0.05 was considered as statistically significant.

## Supplementary Material

loag008_Supplementary_Data

## Data Availability

The data are available from the corresponding authors upon reasonable request.
