# Supplementary material for: Age-specific effects of hemoglobin A1c, blood pressure, and cholesterol levels on incident cardiovascular diseases among adults with diabetes in China: a 10-year prospective cohort study
Source: Life Metab. 2026 Apr 3;5(3):loag008. doi: 10.1093/lifemeta/loag008 (PMC13134377; doi:10.1093/lifemeta/loag008)
Supplement: loag008_Supplementary_Data [file loag008_supplementary_data.zip › 15-Apr-2026_080644_Supplementary_Information--Clear.docx]

**SUPPLEMENTAL MATERIAL**

**Table of Contents:**

**Supplementary Table S1** Baseline clinical characteristics of included and excluded participants (due to missing follow-up or missing baseline ABC measures). 3

**Supplementary Table S2** Age-group distribution of included and excluded participants (due to missing follow-up or missing baseline ABC measures). 4

**Supplementary Table S3** Sensitivity analysis: analyses restricted to participants with diabetes receiving glucose-lowering medications 5

**Supplementary Table S4** Sensitivity analysis: analyses without age adjustment within the age-stratified models 6

**Supplementary Table S5** Sensitivity analysis using Fine−Gray subdistribution hazards models: Associations of risk factors with incident CVD across age strata. 7

**Supplementary Table S6** Sensitivity analysis: exclusion of participants with a history of cancer and those with reduced renal function (eGFR < 60 mL/min/1.73 m^2^) at baseline 8

**Supplementary Table S7** Sensitivity analysis: models further adjusted for baseline presence of comorbidities, renal function (eGFR), and healthcare access. 9

**Supplementary Table S8** ICD-10 Codes used in the 4C study for cardiovascular outcomes. 10

**Supplementary Figure S1** Restricted cubic spline associations of HbA1c, systolic blood pressure, and LDL-C with CVD risk in the total population and across age groups. 11

**Supplementary Table S1** Baseline clinical characteristics of included and excluded participants (due to missing follow-up or missing baseline ABC measures).

|  | Included  (*n* = 36583) | Excluded^∗^  (*n* = 8416) |
| --- | --- | --- |
| Age, year | 60.04 ± 8.87 | 60.00 ± 8.83 |
| Male, *n* (%) | 13941 (38.1%) | 3127 (37.2%) |
| Current smoking, *n* (%) | 5082 (14.6%) | 1146 (14.1%) |
| Current drinking, *n* (%) | 3711 (10.8%) | 656 (8.2%) |
| FPG (mmol/L) | 7.78 ± 2.59 | 7.52 ± 2.49 |
| 2h-PG (mmol/L) | 13.32 ± 4.94 | 12.63 ± 4.58 |
| Hemoglobin A1c (%) | 7.15 ± 1.57 | 7.13 ± 1.46 |
| SBP (mmHg) | 140.41 ± 21.31 | 136.27 ± 18.62 |
| DBP (mmHg) | 80.50 ± 11.37 | 76.86 ± 10.42 |
| LDL-cholesterol (mmol/L) | 2.96 ± 0.91 | 3.13 ± 0.92 |
| HDL-cholesterol (mmol/L) | 1.27 ± 0.34 | 1.30 ± 0.35 |
| Total cholesterol (mmol/L) | 5.12 ± 1.21 | 5.21 ± 1.18 |
| Triglycerides (mmol/L) | 1.61 (1.12−2.37) | 1.54 (1.08−2.23) |
| BMI (kg/m^2^) | 25.74 ± 3.77 | 25.70 ± 3.68 |
| Anti-hypertensive medication, *n* (%) | 6415 (17.5%) | 2054 (24.4%) |
| Lipid-lowering medication, *n* (%) | 428 (1.2%) | 253 (3.0%) |
| Glucose-lowering medication, *n* (%) | 11457 (31.3%) | 2543 (30.2%) |

Values are mean (SD) or *n* (%).

^∗^The excluded group (*n* = 8416) contained 7601 participants for missing follow-up and 815 for missing baseline ABC measures. BMI, body mass index; FPG, fasting plasma glucose; 2h-PG, 2-h post-load glucose; SBP, systolic blood pressure; DBP, diastolic blood pressure.

**Supplementary Table S2** Age-group distribution of included and excluded participants (due to missing follow-up or missing baseline ABC measures).

| Age group | Included (%)  (*n* = 36583) | Excluded^∗^ (%)  (*n* = 8416) |
| --- | --- | --- |
| Young (< 55 years) | 10399 (28.4%) | 2550 (30.3%) |
| Middle aged (55 to < 65 years) | 15476 (42.3%) | 3582 (42.6%) |
| Old (65 to < 75 years) | 9084 (24.8%) | 1794 (21.3%) |
| Elderly (≥ 75 years) | 1624 (4.4%) | 490 (5.8%) |

^∗^The excluded group (*n* = 8416) contained 7601 participants for missing follow up and 815 for missing baseline ABC measures.

**Supplementary Table S3** Sensitivity analysis: associations of risk factors with incident CVD across age strata in Chinese individuals with diabetes receiving glucose-lowering medications^*^.

|  |  | Age group | | | | *P*_interaction_ |
| --- | --- | --- | --- | --- | --- | --- |
|  | Total (*n* = 11457) | Young (< 55 years; *n* = 2669) | Middle aged (55 to < 65 years; *n* = 4911) | Old (65 to < 75 years; *n* = 3306) | Elderly (≥ 75 years; *n* = 571) |  |
| HbA1c (%) | | | | | | |
| < 7 | 1.00 (Ref) | 1.00 (Ref) | 1.00 (Ref) | 1.00 (Ref) | 1.00 (Ref) | 0.129 |
| 7 to < 8 | 1.09 (0.97−1.24) | 0.95 (0.64−1.43) | 1.24 (1.02−1.51) | 0.98 (0.81−1.19) | 1.17 (0.78−1.76) |  |
| 8 to < 9 | 1.33 (1.15−1.53) | 1.30 (0.84−2.02) | 1.53 (1.22−1.90) | 1.22 (0.97−1.54) | 0.95 (0.56−1.62) |  |
| ≥ 9 | 1.64 (1.45−1.86) | 2.11 (1.51−2.95) | 1.75 (1.43−2.13) | 1.37 (1.10−1.70) | 1.68 (1.12−2.51) |  |
| SBP (mmHg) | | | | | | |
| < 120 | 1.00 (Ref) | 1.00 (Ref) | 1.00 (Ref) | 1.00 (Ref) | 1.00 (Ref) | < 0.001 |
| 120 to < 130 | 1.21 (0.99−1.47) | 1.55 (0.97−2.50) | 1.14 (0.85−1.54) | 1.14 (0.80−1.63) | 0.89 (0.38−2.07) |  |
| 130 to < 140 | 1.18 (0.97−1.42) | 1.24 (0.75−2.04) | 1.08 (0.80−1.45) | 1.13 (0.82−1.58) | 1.27 (0.62−2.63) |  |
| ≥ 140 | 1.58 (1.33−1.86) | 2.31 (1.51−3.53) | 1.54 (1.20−1.97) | 1.40 (1.04−1.87) | 1.18 (0.61−2.27) |  |
| LDL-C (mmol/L) | | | | | | |
| < 2.6 | 1.00 (Ref) | 1.00 (Ref) | 1.00 (Ref) | 1.00 (Ref) | 1.00 (Ref) | 0.040 |
| 2.6 to < 3.4 | 1.03 (0.92−1.15) | 1.22 (0.88−1.68) | 0.91 (0.76−1.09) | 1.04 (0.86−1.25) | 1.34 (0.92−1.95) |  |
| 3.4 to < 4.1 | 1.23 (1.08−1.41) | 1.23 (0.83−1.83) | 1.15 (0.94−1.42) | 1.43 (1.16−1.77) | 0.98 (0.62−1.56) |  |
| ≥ 4.1 | 1.24 (1.05−1.46) | 1.98 (1.25−3.13) | 1.42 (1.10−1.84) | 1.05 (0.79−1.38) | 0.74 (0.40−1.37) |  |

^*^Adjusted for age, sex, body-mass index, current smoking, current drinking, educational attainment, receiving lipid-lowering medication, and anti-hypertensive medication at baseline. *P* values for the interaction between age group and each individual risk factor are shown to evaluate variations in the associations between individual risk factors and incident CVD across different age groups.

**Supplementary Table S4** Sensitivity analysis: associations of risk factors with incident CVD without adjusting age in the analysis across age strata^*^.

|  |  | **Age group** | | | | *P*_interaction_ |
| --- | --- | --- | --- | --- | --- | --- |
|  | Total (*n* = 36583) | Young (< 55 years; *n* = 10399) | Middle aged (55 to < 65 years; *n* = 15476) | Old (65 to < 75 years; *n* = 9084) | Elderly (≥ 75 years; *n* = 1624) |  |
| HbA1c (%) | | | | | | |
| < 7 | 1.00 (Ref) | 1.00 (Ref) | 1.00 (Ref) | 1.00 (Ref) | 1.00 (Ref) | < 0.001 |
| 7 to < 8 | 1.16 (1.07−1.25) | 1.04 (0.82−1.32) | 1.26 (1.11−1.43) | 1.08 (0.95−1.23) | 1.03 (0.79−1.34) |  |
| 8 to < 9 | 1.36 (1.23−1.52) | 1.38 (1.02−1.85) | 1.55 (1.32−1.83) | 1.28 (1.07−1.52) | 1.19 (0.81−1.74) |  |
| ≥ 9 | 1.60 (1.46−1.74) | 2.41 (1.96−2.96) | 1.77 (1.54−2.03) | 1.42 (1.20−1.67) | 1.50 (1.12−2.01) |  |
| SBP (mmHg) | | | | | | |
| < 120 | 1.00 (Ref) | 1.00 (Ref) | 1.00 (Ref) | 1.00 (Ref) | 1.00 (Ref) | < 0.001 |
| 120 to < 130 | 1.28 (1.13−1.45) | 1.40 (1.04−1.88) | 1.15 (0.95−1.40) | 1.08 (0.86−1.36) | 1.26 (0.79−2.02) |  |
| 130 to < 140 | 1.38 (1.22−1.56) | 1.53 (1.15−2.05) | 1.12 (0.92−1.36) | 1.11 (0.90−1.38) | 1.22 (0.78−1.89) |  |
| ≥ 140 | 2.14 (1.93−2.38) | 2.54 (1.97−3.27) | 1.76 (1.50−2.07) | 1.50 (1.24−1.82) | 1.24 (0.84−1.84) |  |
| LDL-C (mmol/L) | | | | | | |
| < 2.6 | 1.00 (Ref) | 1.00 (Ref) | 1.00 (Ref) | 1.00 (Ref) | 1.00 (Ref) | 0.013 |
| 2.6 to < 3.4 | 1.12 (1.04−1.20) | 1.30 (1.07−1.58) | 1.04 (0.92−1.17) | 1.07 (0.95−1.20) | 1.24 (0.97−1.58) |  |
| 3.4 to < 4.1 | 1.34 (1.23−1.46) | 1.44 (1.14−1.81) | 1.28 (1.12−1.46) | 1.26 (1.09−1.44) | 1.30 (0.99−1.71) |  |
| ≥ 4.1 | 1.42 (1.28−1.58) | 1.90 (1.45−2.48) | 1.40 (1.19−1.65) | 1.17 (0.98−1.39) | 1.15 (0.82−1.61) |  |

^*^Adjusted for sex, body-mass index, current smoking, current drinking, educational attainment, receiving glucose-lowering medication, lipid-lowering medication, and anti-hypertensive medication at baseline. And age was not adjusted in the analysis across age strata. *P* values for the interaction between age group and each individual risk factor are shown to evaluate variations in the associations between individual risk factors and incident CVD across different age groups.

**Supplementary Table S5** Sensitivity analysis using Fine−Gray subdistribution hazards models: associations of risk factors with incident CVD across age strata^*^.

|  |  | Age group | | | | *P*_interaction_ |
| --- | --- | --- | --- | --- | --- | --- |
|  | Total (*n* = 36583) | Young (< 55 years; *n* = 10399) | Middle aged (55 to < 65 years; *n* = 15476) | Old (65 to < 75 years; *n* = 9084) | Elderly (≥ 75 years; *n* = 1624) |  |
| HbA1c (%) | | | | | | |
| < 7 | 1.00 (Ref) | 1.00 (Ref) | 1.00 (Ref) | 1.00 (Ref) | 1.00 (Ref) | < 0.001 |
| 7 to < 8 | 1.14 (1.05−1.23) | 1.02 (0.80−1.30) | 1.26 (1.11−1.43) | 1.06 (0.93−1.20) | 1.04 (0.79−1.36) |  |
| 8 to < 9 | 1.38 (1.24−1.54) | 1.38 (1.02−1.85) | 1.56 (1.32−1.83) | 1.25 (1.05−1.49) | 1.24 (0.84−1.85) |  |
| ≥ 9 | 1.69 (1.55−1.85) | 2.40 (1.95−2.95) | 1.76 (1.53−2.03) | 1.38 (1.17−1.63) | 1.49 (1.11−1.98) |  |
| SBP (mmHg) | | | | | | |
| < 120 | 1.00 (Ref) | 1.00 (Ref) | 1.00 (Ref) | 1.00 (Ref) | 1.00 (Ref) | < 0.001 |
| 120 to < 130 | 1.21 (1.06−1.37) | 1.39 (1.04−1.86) | 1.15 (0.94−1.39) | 1.09 (0.86−1.37) | 1.33 (0.83−2.13) |  |
| 130 to < 140 | 1.22 (1.08−1.38) | 1.51 (1.13−2.01) | 1.10 (0.91−1.33) | 1.12 (0.90−1.39) | 1.25 (0.81−1.92) |  |
| ≥ 140 | 1.72 (1.54−1.91) | 2.41 (1.87−3.10) | 1.71 (1.45−2.01) | 1.49 (1.23−1.80) | 1.27 (0.86−1.86) |  |
| LDL-C (mmol/L) | | | | | | |
| < 2.6 | 1.00 (Ref) | 1.00 (Ref) | 1.00 (Ref) | 1.00 (Ref) | 1.00 (Ref) | 0.010 |
| 2.6 to < 3.4 | 1.11 (1.03−1.19) | 1.29 (1.06−1.58) | 1.05 (0.93−1.18) | 1.08 (0.95−1.21) | 1.25 (0.98−1.59) |  |
| 3.4 to < 4.1 | 1.29 (1.18−1.40) | 1.38 (1.09−1.75) | 1.28 (1.12−1.46) | 1.26 (1.10−1.45) | 1.32 (1.01−1.74) |  |
| ≥ 4.1 | 1.33 (1.20−1.48) | 1.83 (1.39−2.39) | 1.40 (1.18−1.65) | 1.19 (1.00−1.41) | 1.12 (0.80−1.58) |  |

^*^Adjusted for age, sex, body-mass index, current smoking, current drinking, educational attainment, receiving glucose-lowering medication, lipid-lowering medication, and anti-hypertensive medication at baseline. *P* values for the interaction between age group and each individual risk factor are shown to evaluate variations in the associations between individual risk factors and incident CVD across different age groups.

**Supplementary Table S6** Sensitivity analysis: associations of risk factors with incident CVD across age strata in Chinese individuals without cancer and reduced renal function (eGFR <60 mL/min/1.73 m^2^) at baseline^*^.

|  |  | Age group | | | | *P*_interaction_ |
| --- | --- | --- | --- | --- | --- | --- |
|  | Total (*n* = 34540) | Young (< 55 years; *n* = 10153) | Middle aged (55 to < 65 years; *n* = 14796) | Old (65 to < 75 years; *n* = 8259) | Elderly (≥ 75 years; *n* = 1332) |  |
| HbA1c (%) | | | | | | |
| < 7 | 1.00 (Ref) | 1.00 (Ref) | 1.00 (Ref) | 1.00 (Ref) | 1.00 (Ref) | < 0.001 |
| 7 to < 8 | 1.14 (1.05−1.24) | 1.02 (0.79−1.30) | 1.28 (1.12−1.45) | 1.06 (0.93−1.22) | 1.07 (0.80−1.44) |  |
| 8 to < 9 | 1.40 (1.25−1.57) | 1.45 (1.08−1.95) | 1.59 (1.34−1.87) | 1.24 (1.03−1.50) | 1.18 (0.77−1.81) |  |
| ≥ 9 | 1.78 (1.63−1.96) | 2.49 (2.02−3.07) | 1.80 (1.56−2.08) | 1.48 (1.25−1.76) | 1.62 (1.15−2.27) |  |
| SBP (mmHg) | | | | | | |
| < 120 | 1.00 (Ref) | 1.00 (Ref) | 1.00 (Ref) | 1.00 (Ref) | 1.00 (Ref) | < 0.001 |
| 120 to < 130 | 1.19 (1.04−1.36) | 1.39 (1.03−1.88) | 1.15 (0.94−1.41) | 1.04 (0.82−1.32) | 1.26 (0.73−2.16) |  |
| 130 to < 140 | 1.19 (1.05−1.36) | 1.54 (1.15−2.08) | 1.11 (0.91−1.35) | 1.01 (0.80−1.26) | 1.32 (0.80−2.18) |  |
| ≥ 140 | 1.71 (1.53−1.91) | 2.43 (1.87−3.15) | 1.71 (1.45−2.01) | 1.42 (1.17−1.73) | 1.25 (0.79−1.97) |  |
| LDL-C (mmol/L) | | | | | | |
| < 2.6 | 1.00 (Ref) | 1.00 (Ref) | 1.00 (Ref) | 1.00 (Ref) | 1.00 (Ref) | 0.011 |
| 2.6 to < 3.4 | 1.09 (1.01−1.18) | 1.24 (1.02−1.52) | 1.05 (0.93−1.18) | 1.07 (0.94−1.22) | 1.22 (0.93−1.59) |  |
| 3.4 to < 4.1 | 1.29 (1.18−1.40) | 1.37 (1.08−1.73) | 1.29 (1.12−1.47) | 1.26 (1.09−1.47) | 1.27 (0.94−1.72) |  |
| ≥ 4.1 | 1.32 (1.18−1.47) | 1.79 (1.36−2.35) | 1.38 (1.17−1.64) | 1.16 (0.97−1.40) | 1.05 (0.71−1.54) |  |

^*^Adjusted for age, sex, body-mass index, current smoking, current drinking, educational attainment, receiving glucose-lowering medication, lipid-lowering medication, and anti-hypertensive medication at baseline. *P* values for the interaction between age group and each individual risk factor are shown to evaluate variations in the associations between individual risk factors and incident CVD across different age groups.

**Supplementary Table S7** Sensitivity analysis: associations of risk factors with incident CVD further adjusting for baseline presence of comorbidities, renal function (eGFR), and healthcare access across age strata^*^.

|  |  | Age group | | | | *P*_interaction_ |
| --- | --- | --- | --- | --- | --- | --- |
|  | Total (*n* = 36583) | Young (< 55 years; *n* = 10399) | Middle aged (55 to < 65 years; *n* = 15476) | Old (65 to < 75 years; *n* = 9084) | Elderly (≥ 75 years; *n* = 1624) |  |
| HbA1c (%) | | | | | | |
| < 7 | 1.00 (Ref) | 1.00 (Ref) | 1.00 (Ref) | 1.00 (Ref) | 1.00 (Ref) | < 0.001 |
| 7 to < 8 | 1.16 (1.06−1.25) | 1.05 (0.82−1.34) | 1.28 (1.13−1.46) | 1.08 (0.95−1.23) | 1.08 (0.82−1.42) |  |
| 8 to < 9 | 1.39 (1.25−1.55) | 1.40 (1.03−1.90) | 1.55 (1.31−1.83) | 1.25 (1.05−1.50) | 1.32 (0.90−1.95) |  |
| ≥ 9 | 1.74 (1.58−1.90) | 2.39 (1.94−2.95) | 1.76 (1.52−2.03) | 1.44 (1.22−1.70) | 1.61 (1.19−2.18) |  |
| SBP (mmHg) | | | | | | |
| < 120 | 1.00 (Ref) | 1.00 (Ref) | 1.00 (Ref) | 1.00 (Ref) | 1.00 (Ref) | < 0.001 |
| 120 to < 130 | 1.19 (1.04−1.35) | 1.36 (1.01−1.83) | 1.14 (0.93−1.39) | 1.06 (0.84−1.34) | 1.24 (0.77−1.99) |  |
| 130 to < 140 | 1.16 (1.03−1.32) | 1.50 (1.12−2.02) | 1.05 (0.86−1.28) | 1.02 (0.82−1.28) | 1.19 (0.76−1.85) |  |
| ≥ 140 | 1.66 (1.49−1.85) | 2.31 (1.78−2.99) | 1.67 (1.42−1.97) | 1.40 (1.15−1.70) | 1.12 (0.75−1.67) |  |
| LDL-C (mmol/L) | | | | | | |
| < 2.6 | 1.00 (Ref) | 1.00 (Ref) | 1.00 (Ref) | 1.00 (Ref) | 1.00 (Ref) | 0.002 |
| 2.6 to < 3.4 | 1.08 (1.00−1.17) | 1.28 (1.04−1.57) | 1.04 (0.93−1.18) | 1.02 (0.90−1.16) | 1.17 (0.91−1.51) |  |
| 3.4 to < 4.1 | 1.26 (1.16−1.38) | 1.43 (1.13−1.81) | 1.27 (1.11−1.46) | 1.21 (1.05−1.40) | 1.16 (0.87−1.54) |  |
| ≥ 4.1 | 1.27 (1.14−1.41) | 1.82 (1.38−2.40) | 1.35 (1.13−1.60) | 1.07 (0.89−1.28) | 1.05 (0.74−1.49) |  |

^*^Adjusted for age, sex, body-mass index, current smoking, current drinking, educational attainment, receiving glucose-lowering medication, lipid-lowering medication, anti-hypertensive medication, presence of comorbidities, renal function (eGFR), and healthcare access at baseline. *P* values for the interaction between age group and each individual risk factor are shown to evaluate variations in the associations between individual risk factors and incident CVD across different age groups.

**Supplementary Table S8** ICD-10 Codes used in the 4C study for cardiovascular outcomes.

| **Cardiovascular disease outcomes** | **ICD-10 codes** |
| --- | --- |
| **Non-fatal myocardial infarction** | I21–I22 |
| **Non-fatal stroke** | I60–I64, I69 |
| **Cardiovascular death** | I00–I99 |


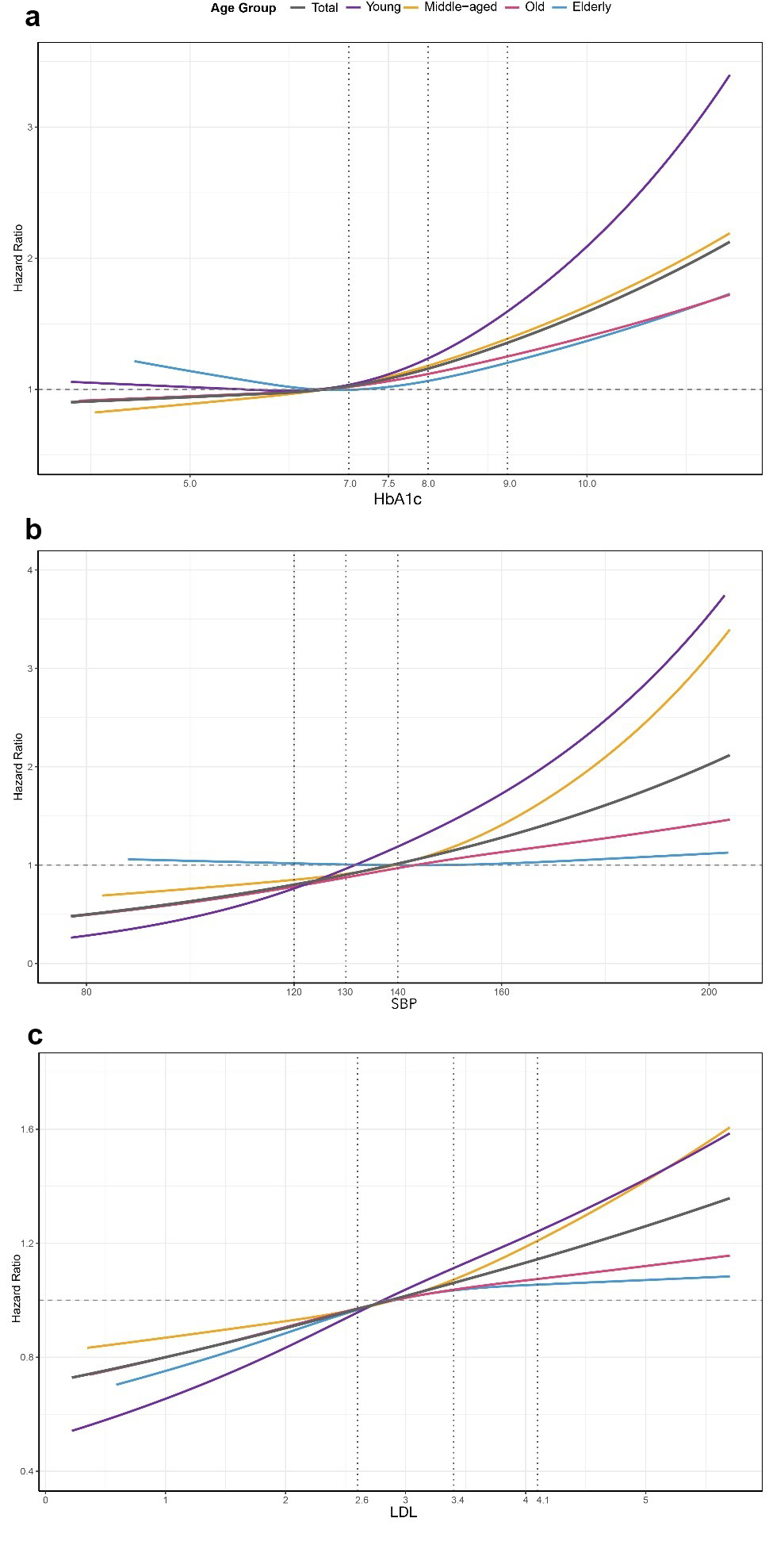


**Supplementary Figure S1** Restricted cubic spline associations of (a) HbA1c, (b) systolic blood pressure, and (c) LDL-C with CVD risk in the total population and across age groups. Models were adjusted for sex, age, body-mass index, current smoking, current drinking, educational attainment, receiving glucose-lowering medication, lipid-lowering medication, and anti-hypertensive medication at baseline.
